# A theory-based online health behaviour intervention for new university students (U@Uni:LifeGuide): results from a repeat randomized controlled trial

**DOI:** 10.1186/s13063-015-1092-4

**Published:** 2015-12-07

**Authors:** David Cameron, Tracy Epton, Paul Norman, Paschal Sheeran, Peter R. Harris, Thomas L. Webb, Steven A. Julious, Alan Brennan, Chloe Thomas, Andrea Petroczi, Declan Naughton, Iltaf Shah

**Affiliations:** Department of Psychology, University of Sheffield, Western Bank, Sheffield, S10 2TP UK; School of Psychological Science, University of Manchester, Oxford Road, Manchester, M13 9PL UK; Psychology Department, University of North Carolina, 323 Davie Hall, Chapel Hill, NC 27599-3270 USA; School of Psychology, University of Sussex, Falmer, Brighton, BN1 9QH UK; School of Health and Related Research, University of Sheffield, Regent Court, Sheffield, S1 4DA UK; School of Life Sciences, Pharmacy and Chemistry, Kingston University, Penrhyn Road, Kingston upon Thames, KT1 2EE UK

**Keywords:** Binge drinking, Diet, Exercise, Implementation intentions, Internet, Self-affirmation, Smoking, Students, Theory of planned behaviour, Young people

## Abstract

**Background:**

This paper reports the results of a repeat trial assessing the effectiveness of an online theory-based intervention to promote healthy lifestyle behaviours in new university students. The original trial found that the intervention reduced the number of smokers at 6-month follow-up compared with the control condition, but had non-significant effects on the other targeted health behaviours. However, the original trial suffered from low levels of engagement, which the repeat trial sought to rectify.

**Methods:**

Three weeks before staring university, all incoming undergraduate students at a large university in the UK were sent an email inviting them to participate in the study. After completing a baseline questionnaire, participants were randomly allocated to intervention or control conditions. The intervention consisted of a self-affirmation manipulation, health messages based on the theory of planned behaviour and implementation intention tasks. Participants were followed-up 1 and 6 months after starting university. The primary outcome measures were portions of fruit and vegetables consumed, physical activity levels, units of alcohol consumed and smoking status at 6-month follow-up.

**Results:**

The study recruited 2,621 students (intervention *n* = 1346, control *n* = 1275), of whom 1495 completed at least one follow-up (intervention *n* = 696, control *n* = 799). Intention-to-treat analyses indicated that the intervention had a non-significant effect on the primary outcomes, although the effect of the intervention on fruit and vegetable intake was significant in the per-protocol analyses. Secondary analyses revealed that the intervention had significant effects on having smoked at university (self-report) and on a biochemical marker of alcohol use.

**Conclusions:**

Despite successfully increasing levels of engagement, the intervention did not have a significant effect on the primary outcome measures. The relatively weak effects of the intervention, found in both the original and repeat trials, may be due to the focus on multiple versus single health behaviours. Future interventions targeting the health behaviour of new university students should therefore focus on single health behaviours.

**Trial registration:**

Current Controlled Trials ISRCTN07407344.

## Background

The performance of health-promoting behaviours (e.g., eating fruit and vegetables, engaging in regular exercise), coupled with the avoidance of health-risk behaviours (e.g., excessive alcohol consumption, smoking), is important in reducing the risk of developing serious health problems, such as cancer, cardiovascular disease, obesity and type 2 diabetes [1]. A recent survey in England indicated that among 16–24 year olds, only 17 % of men and 19 % of women ate at least five portions of fruit and vegetables per day, 83 % of men but only 57 % of women met recommended levels of weekly physical activity, 27 % of men and 19 % of women consumed more than double the recommended daily limit of alcohol in the previous week and 27 % of men and 19 % of women were current smokers [2, 3]. Given that there is evidence of clustering of health behaviours [4, 5], it is likely that many young people engage in a number of health-compromising behaviours, thereby placing themselves at increased risk of developing serious health problems. There is therefore a clear need for interventions that target multiple health behaviours in young people. Encouragingly, there is some evidence that such interventions can have positive effects on health behaviour [6–8].

An earlier trial [9] tested the efficacy of a theory-based online intervention (U@Uni) targeting four health behaviours (fruit and vegetable intake, physical activity, alcohol consumption, and smoking) during the transition from school to university. Such life transitions are ideal opportunities to intervene as they represent ‘teachable’ moments; times when people’s social environments and supporting cues for behaviour are in a state of flux and people are therefore more amenable to change [10, 11]. Moreover, given that more than 500,000 students and over 30 % of 18 year olds in England enter higher education each year [12], interventions delivered during this transition have the potential to reach a large number of young people. The intervention used three theory-based techniques to target the four health behaviours. First, a self-affirmation manipulation was used to reduce defensive processing of health messages [13]. Second, theory-based messages were developed through formative research to target the key beliefs underlying the four health behaviours, in order to increase young people’s motivation to engage in healthy behaviours [14]. Third, implementation intention tasks were included to help translate good intentions into healthy behaviour [15].

The intervention had a significant effect on smoking, with fewer current smokers at follow-up in the intervention than in the control condition, although the intervention did not significantly affect the other three primary outcomes (i.e., fruit and vegetable intake, physical activity, alcohol consumption) [16]. Despite these largely non-significant effects, the health economic modelling revealed that rolling out the intervention to other universities would be likely to be cost-effective, primarily because of the low cost of the intervention and the impact of reduced smoking on future health outcomes [17].

Unfortunately, the trial was compromised by a number of limitations, which resulted in low levels of engagement with the intervention. Only 52 % of participants allocated to the intervention condition completed the self-affirmation task, only 35 % accessed the health messages and only 1 % formed an implementation intention. As a result, it is difficult to determine whether the non-significant results are due to failure of theory or failure of intervention fidelity caused by low engagement. In addition, low engagement is also likely to lead to an inaccurate estimate of the effect of the intervention on the health behaviours, which an expected value of information analysis identified as an important driver of decision uncertainty in the health economic modelling [17].

There were three potential reasons for low engagement. First, the baseline questionnaire was time-consuming to complete (approximately 20 minutes) due to the large number of items needed to assess the primary and secondary outcome variables. Having completed the questionnaire, many participants may have been fatigued and less inclined to proceed to, and engage with, the intervention. Second, the bespoke software platform that was developed to deliver the intervention had a number of technical glitches, meaning that participants’ experience of completing the baseline questionnaire and engaging with the intervention was suboptimal. In particular, what was intended as a seamless process was experienced as a series of discrete steps with subsequent drop-outs at each step. For example, after completing the baseline questionnaire, participants in the intervention condition were directed to the U@Uni log-in page where they had to enter some registration details before completing the self-affirmation manipulation. After completing the self-affirmation manipulation, participants had to log in again to access the intervention material, and many failed to do so. Third, participants in the intervention condition had complete control over the amount and type of intervention material that they viewed. For example, participants in the intervention condition could choose which health behaviours and which belief-based messages to view and whether or not to make plans. This is also likely to have reduced engagement, as participants could simply choose not to view any messages or make any plans.

With these limitations in mind, a repeat trial was conducted with a number of changes designed to increase engagement with the intervention and provide a more accurate estimate of the efficacy of the intervention. First, the baseline questionnaire was shortened. In particular, shorter and simpler measures of fruit and vegetable intake and alcohol consumption were included, and some secondary outcome measures (e.g., self-efficacy, perceived control) were removed. Second, the intervention was delivered using the LifeGuide open-source software platform [18]. LifeGuide has been specifically designed for researchers to develop, deliver and evaluate online health behaviour interventions. It allows participants to complete baseline measures, be randomly allocated to conditions and access intervention material, all within the same website. As a result, participants experience the various tasks as seamless, with reduced opportunity to exit between tasks. Third, the key content of the intervention was delivered in a more structured format so that participants could quickly access health messages and make plans for all four health behaviours. In particular, participants in the intervention condition worked through four short modules that required them to read at least one belief-based message and make at least one plan for each health behaviour, before gaining access to the full intervention website.

This paper reports the results of the repeat randomized controlled trial of a theory-based online health behaviour intervention delivered during the transition from school to university. The primary research question was whether the intervention produces significant changes in the health behaviours of new students (i.e., fruit and vegetable intake, physical activity, alcohol consumption and smoking status) at a 6-month follow-up. Additional research questions focused on whether the intervention (i) changes theory of planned behaviour variables (and whether these changes mediate the effect of the intervention on the health behaviours), (ii) enhances health status, (iii) reduces health service usage, (iv) reduces recreational drug use and (v) reduces body mass index (BMI).

## Methods

### Participants and procedure

**Fig. 1:**
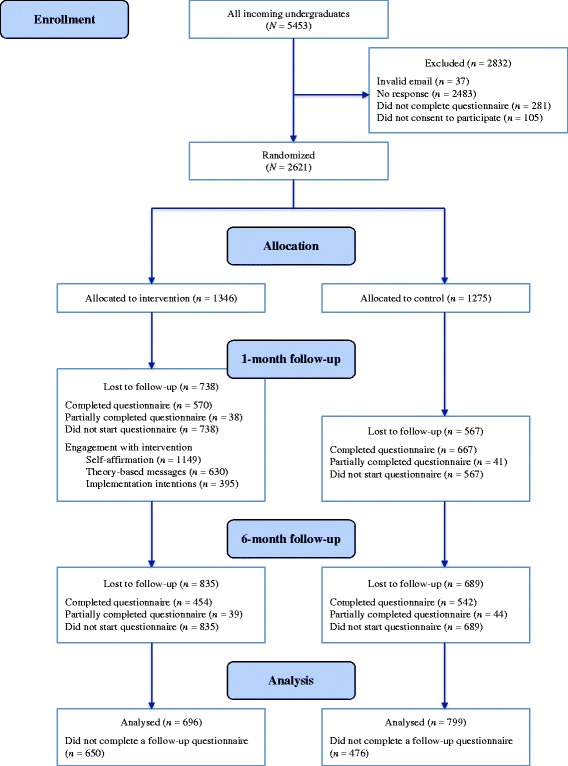
Flow of participants through the trial

Three weeks before starting university (in September 2013), all incoming undergraduate students to the University of Sheffield (*N* = 5,453) were sent an email inviting them to take part in the study, with a link to an online questionnaire containing measures of demographics, health status, intentions, and health behaviour. There were no exclusion criteria. Participants indicated their consent to participate by clicking a button on the first page before they were permitted to proceed to the rest of the questionnaire. Participants (*N* = 2,621; mean age = 18.80 years; 55 % women) then completed the baseline questionnaire and were randomly allocated to the intervention (*n* = 1,346) and control (*n* = 1,275) conditions using the random function on LifeGuide [18]. Figure 1 shows the flow of participants through the trial and Table 1 provides details of the sample at baseline in terms of demographics and the primary and secondary outcomes. In addition, the proportions of the sample meeting guidelines for the four health behaviours at baseline are reported in Table 2, along with data for 16–24-year-olds from the Health Survey for England [2, 3].

**Table 1:** Characteristics of the sample at baseline

Variable		Control			Intervention		
		% or mean	Standard deviation	*n*	% or mean	Standard deviation	*n*
Demographics							
Nationality	UK	75.37	–	961	78.16	–	1052
	Non-UK	24.63	–	314	21.84	–	294
Ethnicity	White British	68.67	–	835	71.65	–	930
	White other	7.40	–	90	6.32	–	82
	Mixed	3.87	–	47	3.70	–	48
	Asian and Asian British	5.10	–	62	5.32	–	69
	Black and Black British	206	–	25	2.77	–	36
	Chinese	10.20	–	124	8.24	–	107
	Other	2.71	–	33	2.00	–	26
Sex	Female	54.87	–	698	55.81	–	749
	Male	45.13	–	574	44.19	–	593
Age		18.89	2.68	1274	18.73	2.01	1340
Fruit and vegetable intake							
Portions per day		4.48	2.21	1267	4.49	2.34	1344
Physical activity							
Metabolic equivalent of task per week		3665.30	3518.61	1273	3510.02	3276.63	1343
Alcohol consumption							
Total units the in previous 7 days		6.77	9.32	1271	7.15	9.72	1344
Number of days binge drinking in the last 7 days (drinkers only)		0.52	0.80	833	0.56	0.82	872
Biochemical marker of alcohol consumption (fatty acid ethyl esters)		3.24	4.09	123	2.60	3.33	90
Smoking							
Has smoked		42.89	–	1175	44.31	–	1248
Current smoker		3.49	–	1175	2.80	–	1248
Cigarettes smoked per week (smokers only)		49.05	38.93	41	53.60	45.95	35
Smoking marker (cotinine)		0.48	0.44	123	0.44	0.41	90
Smoking marker (nicotine)		3.89	10.32	123	6.52	17.83	90
Other outcomes							
EQ-5D-3 L							
Health index score from EQ-5D-3 L (visual analogue scale)		0.94	0.12	1249	0.94	0.12	1327
Health index score from EQ-5D-3 L (time trade off)		0.95	0.11	1249	0.95	0.11	1327
EQ-5D-3 L visual analogue scale		83.26	12.12	1256	82.01	13.77	1326
Recreational drugs							
Have taken recreational drugs (single sample count method)		19.11	7.21	1275	17.84	6.79	1336
Have taken recreational drugs (biochemical marker)		24.39	–	123	24.44	–	123
Body mass index							
Self-report		21.78	3.69	1206	21.95	3.88	1274
Objective		21.67	3.55	123	21.5	3.39	90
Social cognition variables							
Fruit and vegetable intention		4.78	1.59	1150	4.76	1.58	1258
Physical activity intention		5.72	1.38	1156	5.69	1.43	1263
Binge drinking intention		3.33	1.90	1156	3.32	1.92	1249
Smoking intention		1.51	1.29	1173	1.46	1.20	1269

**Table 2:** Percentages of men and women meeting health behaviour guidelines in the baseline sample and among 16–24 years in England [2, 3]

	Baseline sample		Health Survey for England data (16–24-year-olds)	
Variable	Men	Women	Men	Women
Fruit and vegetable intake				
Five or more portions per day	41	44	17	19
Physical activity				
150 minutes per week	83	61	83	57
Alcohol consumption				
Not more than 21 (men) or 14 (women) units per week	88	91	80	83
Smoking				
Current non-smoker	3	3	27	19

After completing the baseline questionnaire, participants assigned to the intervention condition were asked to complete a ‘profile’ page that contained the self-affirmation manipulation. They were then directed to complete four short modules on each of the four health behaviours targeted by the intervention that contained theory-based messages and planning exercises. After completing all four modules, intervention participants in the intervention condition had access to the full website with further health messages and links on each of the four targeted health behaviours.

All participants were sent emails inviting them to complete follow-up questionnaires 1 month (October 2013) and 6 months (March 2014) after starting university. Participants were entered into a £100 prize draw as an incentive for completing each questionnaire. In addition, participants completing all three questionnaires received a £10 gift voucher and were entered into a further prize draw for an iPad Mini.

Participants were also sent emails when they started university, inviting them to provide additional data on the biochemical markers of health behaviour. A sample of 213 students (intervention, *n* = 90; control, *n* = 123; mean age = 18.93 years; standard deviation (SD) = 2.76) was recruited at baseline, of whom 133 also provided a hair sample when invited at 6-month follow-up (intervention, *n* = 63; control, *n* = 70).

Ethical approval for the study was obtained from the Department of Psychology Research Ethics Committee at the University of Sheffield.

### Intervention materials

The self-affirmation manipulation was adapted from an existing value-affirmation task [19] and embedded in a ‘profile’ page. Participants were asked to provide details including their name, course, home town and main interests or hobbies, before being presented with a list of eight commonly held personal values (sense of humour, academic achievement, relations with family and friends, social skills, spontaneity, artistic skills or aesthetic appreciation, religion, faith or spirituality, and respect, decency or manners). Participants were asked to select their most important value (or provide their own) and to explain briefly why the value was important to them. The resultant information formed part of the user’s ‘profile’, which was displayed in the banner at the top of all pages of the intervention website that included the participant’s name, the value that they chose and the reason why it was important to them (‘I value X because Y.’).

After completing the self-affirmation manipulation, participants were directed to complete short modules on each of the four targeted health behaviours. Theory-based messages were developed to encourage adequate fruit and vegetable intake and regular exercise, and to discourage binge drinking and smoking. The messages were based on the theory of planned behaviour [20] and developed on the basis of formative work that identified the key behavioural, normative and control beliefs associated with intentions to perform each of the four health behaviours in new university students [14].

Module 1 focused on exercising regularly at university. Participants were presented with a list of topics that targeted key beliefs from the formative research and instructed to choose one (e.g., ‘Exercise improves your fitness’). They were then directed to a webpage containing theory-based messages (i.e., text), videos of students talking about the targeted belief, and links to other related material. After viewing the page, participants had the opportunity to either view another topic or message or proceed to the planner.

The planner helped participants to form implementation intentions by asking them to identify (i) a good opportunity to act on their intentions (e.g., when they have spare time between lectures) and (ii) a suitable response to their identified opportunity (e.g., to go swimming in the university pool) for each of the four targeted health behaviours. Participants were presented with an example of an implementation intention in an if-then format [21] (e.g., ‘IF I am tempted to skip exercising, THEN I will tell myself ‘no excuses’ and remind myself that I will feel great after exercising’). Spaces were provided for participants to make up to three if-then plans by linking an identified opportunity and appropriate response. Participants were presented with their plan and asked to repeat it to themselves several times. A record of the plan was also automatically emailed to the participant.

When participants had finished Module 1, they were presented with the first page of Module 2 (‘Eating fruit and vegetables’), and instructed to work through the module in the same way as for Module 1. Participants then completed Modules 3 (‘Avoiding binge drinking’) and 4 (‘Avoiding smoking’). When all four modules had been completed, participants had access to the full website, containing messages targeting all of the key beliefs from the formative research, links to the planner, saved plans and general health information. All participants completed the modules in the same order as described.

### Measures

The four primary outcome measures were (i) the number of portions of fruit and vegetables consumed per day, (ii) physical activity in the previous week, (iii) alcohol consumption in the previous week, and (iv) smoking status at 6-month follow-up. A range of secondary outcome measures was also assessed as detailed next. Unless indicated, all of the measures were taken at baseline as well as at 1- and 6-month follow-up.

#### Fruit and vegetable intake

Fruit and vegetable intake (portions per day) was measured using a two-item dietary questionnaire [22], which has been validated against biochemical markers (e.g., potassium excretion, urinary potassium:creatinine ratio and plasma concentration of vitamin C). Participants were asked to report the amount of (i) fruit and (ii) vegetables consumed in a typical day (at baseline) and since starting university (at 1 and 6-month follow-up). Reponses to the items were summed to give an estimated total of daily fruit and vegetable consumption.

#### Physical activity

The Short Form of International Physical Activity Questionnaire was used to assess levels of physical activity [23]. This questionnaire has undergone extensive testing across 12 countries and evidence attests to its reliability and validity. Participants were asked to indicate how many times, and for how long, they had engaged in vigorous exercise (defined as ‘activities that take hard physical effort and make you breathe much harder than normal’), moderate exercise (defined as ‘activities that take moderate physical effort and make you breathe somewhat harder than normal’) and walking in the previous 7 days. Responses were converted into ‘metabolic equivalents of task’, to provide a total score representing the total amount of physical activity over the 7 days.

#### Alcohol

Alcohol consumption was assessed using a retrospective 7-day recall drinking diary, in which participants reported the amount of alcohol (units) consumed on each of the previous 7 days [24]. The total number of units of alcohol consumed in the previous week and the number of binge sessions were calculated. The Alcohol Use Disorders Identification Test (AUDIT) [25] was also included at 6-month follow-up, to assess hazardous and harmful patterns of alcohol use.

#### Smoking

Items based on the Health Survey for England [2] were used to assess participants’ current smoking status and the typical number of cigarettes or amount of tobacco that they smoked. In addition, at 1 and 6-month follow-up, participants were asked, ‘Since starting university have you smoked at all (even a puff or just socially)?’

#### Health status

The EQ-5D-3 L [26], a short standardized measure of health status, was used to assess levels of severity (no problems, some or moderate problems, extreme problems) in five domains: mobility, self-care, usual activities, pain or discomfort, and anxiety or depression. The EQ-5D-3 L provides a descriptive profile and a single index value for health status and is recommended as the measure of health-related quality of life for health economic evaluations in the UK [27]. The EQ-5D-3 L was assessed at baseline and 6-month follow-up.

#### Recreational drug use

A single sample count method [28] was used to estimate the prevalence of recreational drug use in the sample. Respondents were asked to indicate the number of ‘yes’ answers (0 or 5, 1, 2, 3, 4) to five questions – four of which have a 50 % population prevalence (e.g., odd or even date of birth) and one of which was about their use of recreational drugs. The position of the sensitive item (i.e., ‘I have used recreational drugs in the last 3 months/since starting university’) was randomized. The single sample count method can be used to provide an estimate of the prevalence of recreational drug use in the sample without being able to identify whether individual participants do or do not use recreational drugs, on the basis that 50 % of the sample should answer ‘yes’ answers to each of the four non-sensitive questions. This method has been shown to encourage accurate reporting of behaviours that are illegal and could be regarded as socially undesirable [29].

#### Body mass index

All participants recorded their height and weight, from which their BMI was calculated. Those participants who provided a hair sample for analysis also had their height and weight measured to provide an objective measure of BMI.

#### Health services usage

Participants were asked to report their use of health services (e.g., general practitioner visits, hospitalizations) at the 6-month follow-up.

#### Social cognitive variables

Single-item measures of social cognitive variables for each behaviour were included. Intentions (e.g., ‘Do you intend to engage in regular exercise at university?’) were measured at all three time points. Affective attitudes (e.g., ‘Engaging in regular exercise at university would be… unpleasant/pleasant’), cognitive attitudes (e.g., ‘Engaging in regular exercise at university would be… harmful/beneficial’), subjective norms (e.g., ‘Most people who are important to me think I should/should not engage in regular exercise at university’), descriptive norms (e.g., ‘Most students will engage in regular exercise at university’), self-efficacy (e.g., ‘If I wanted, I could easily engage in regular exercise at university’), perceived control (e.g., ‘How much control do you have over whether or not you engage in regular exercise at university?’), and planning (e.g., ‘To what extent do you have a clear plan of how to engage in regular exercise at university?’) were assessed at the 1- and 6-month follow-ups.

#### Engagement with the intervention

Engagement with the intervention was measured by identifying whether or not participants (i) completed the self-affirmation task (i.e., profile page), (ii) viewed the theory-based messages in the four modules and (iii) formed implementation intentions for the four health behaviours.

#### Biochemical measures

Hair samples (3 cm long) were liquefied and analyzed for biochemical markers of alcohol consumption, cigarette smoking and recreational drug use. Following extraction procedures, markers of alcohol (fatty acid ethyl esters) and cigarettes (nicotine, cotinine) were quantified using liquid chromatography with tandem mass spectrometric detection. In addition, evidence for recreational drug use was detected by screening for commonly used drugs and their metabolites. These included: amphetamine, 3,4-methylenedioxyamphetamine, 3,4-methylenedioxy-*N*-methylamphetamine, ephedrine, mephedrone, tetrahydrocannabinol, cocaine, heroin, lysergic acid diethylamide, phencyclidine and ketamine. Morphine, codeine, hydromorphone and hydrocodone were treated separately owing to their potential medical use (i.e., as a pain reliever or cough suppressant). A 6430 triple quadruple mass spectrometer (Agilent Technologies UK) was employed, with a dynamic-multiple reaction monitoring-liquid chromatography mass spectrometry method.

### Statistical analysis

The original trial of the U@Uni intervention [16] achieved an initial response rate of 31.34 % to the recruitment emails, and obtained follow-up data for at least one time point from 76.60 % of respondents. *A-priori* sample size calculations for the repeat trial indicated that, assuming the same response and retention rates, a total sample size of approximately 5000 × 0.3134 × 0.7660 = 1200 (i.e., 600 per arm of the trial) would be obtained that would be sufficient to detect a standardized effect size of *d* = 0.20 at a two-sided significance level of 0.0127 with 80 % power. Webb *et al.* [8] reported that the overall effect size of internet-based health behaviour interventions was *d* = 0.16, although this increased for interventions based on the theory of planned behaviour (*d* = 0.36) and using implementation intentions (*d* = 0.25). For the hair analysis, assuming the same response and retention rates as in the original trial, it was estimated that a final sample of 84 would be obtained that would be sufficient to detect a medium effect size of *d* = 0.62 (alpha = 0.05, power = 0.80).

The data analysis plan for the repeat trial was the same as for the original trial [16] and as reported in the study protocol [9]. Analyses assessing intervention effects on the primary outcome variables (i.e., the four targeted health behaviours at 6-month follow-up) were conducted using an intention-to-treat approach (i.e., data were included from all participants who completed at least one follow-up survey). Missing data at 6-months were imputed from the 1-month follow-up data by carrying the last observation forward [30, 31]. A series of analyses of covariance (ANCOVAs) and logistic regression analyses were used to assess the impact of the intervention on performance of the targeted behaviours at 6-month follow-up, controlling for corresponding baseline scores, sex, age and nationality (i.e., UK or non-UK). For primary outcomes, the Bonferroni correction was used to account for multiple tests. Thus, statistical significance was declared if any of the primary endpoints were significant at 0.0127. The primary analyses were repeated without imputing data, as recommended by Altman [32]. Further analyses were conducted to assess the effect of variables that might moderate the effect of the intervention on the primary outcomes, including sex, nationality, ethnicity and engagement with the intervention (per-protocol analyses).

The impact of the intervention on secondary outcomes (i.e., health behaviours at 1 month follow-up, social cognitive variables, health status, recreational drug use, BMI, health services usage, and biochemical measures) was assessed using a similar analysis strategy (i.e., using ANCOVAs and logistic regression analyses that controlled for corresponding baseline scores (where available), sex, age and nationality). No adjustments were made for multiple tests, and intention-to-treat analyses were not performed for the secondary outcomes. Possible adverse consequences of the intervention (i.e., harms) were assessed by considering effects on the primary outcomes and key secondary outcomes (i.e., recreational drug use, AUDIT scores, health status, health services usage).

In addition, analyses were conducted to compare students who did or did not participate in the trial in response to the invitation email on demographics (age, sex, nationality), as well as participants who were randomly allocated to the intervention or control conditions (randomization check), participants who did or did not provide a hair sample, and participants who did or did not complete a follow-up questionnaire (attrition analyses), on the baseline measures using independent sample *t* tests (for continuous variables) and chi-square tests (for categorical variables).

## Results

### Randomization checks

There were no significant differences between participants in the intervention and control conditions on any of the baseline measures (see Table 1).

### Comparison between participants who provided or who did not provide a hair sample

No significant differences were found between participants who provided or did not provide a hair sample on the primary outcome variables at baseline.

### Attrition analyses

Comparing the demographic profile of students who did or did not participate in the trial in response to the invitation email revealed that women were more likely to participate in the trial than men (54.1 % vs. 42.0 %), χ^2^ (1, *N* = 5451) = 79.32, *P* < 0.001, as were non-UK students versus UK students (57.8 % vs. 45.7 %), χ^2^ (1, *N* = 5453) = 49.65, *P* < 0.001. Students who participated in the trial were also slightly younger than those who did not (mean = 18.76, SD = 2.44 vs. mean = 18.95, SD = 2.42), *t* (5451) = 2.96, *P* = 0.003.

Examining attrition after baseline revealed that participants who completed at least one follow-up questionnaire differed from those who did not complete a follow-up questionnaire in nationality, χ^2^ (1, *N* = 2621) = 23.18, *P* < 0.001, ethnicity, χ^2^ (1, *N* = 2514) = 11.39, *P* < 0.001, sex, χ^2^ (1, *N* = 2621) = 33.47, *P* < 0.001, BMI, *t* (2478) = 2.48, *P* = 0.013, and baseline intentions to consume fruit and vegetables, *t* (2406) = 2.38, *P* = 0.017. Completers were more likely to be British, white and female, with a higher BMI and weaker intention to consume fruit and vegetables, than those who did not complete a follow-up questionnaire. In addition, there was a significant difference in drop-out rates between the two conditions, χ^2^ (1, *N* = 2621) = 33.47, *P* < 0.001 (47.5 % intervention, 36.3 % control).

### Primary outcomes

There were no statistically significant differences between the intervention and control conditions on the primary outcomes at 6-month follow-up, although the effect of the intervention on fruit and vegetable intake approached significance: fruit and vegetable intake (*P* = 0.024), physical activity (*P* = 0.932), smoking status (*p* = 0.293), and alcohol consumption (*P* = 0.277). Repeating the primary analyses without data imputation produced consistent results.

The effect sizes found in the repeat trial were comparable to those found in the original trial (see Table 3) for fruit and vegetable intake, *Q*(1) = 2.93, *P* = 0.087, physical activity, *Q*(1) = 0.00015, *P* = 0.990, alcohol consumption, *Q*(1) = 0.25, *P* = 0.619, and smoking status, *Q*(1) = 3.46, *P* = 0.063. However, two of the differences approached significance. A marginally larger effect size was found for fruit and vegetable intake in the repeat trial (*d* = 0.12) than in the original trial (*d* = −0.02), whereas a marginally larger effect size was found for smoking status in the original trial (*d* = 0.25) than in the repeat trial (*d* = 0.10).

**Table 3:** Estimated marginal means, percentages, sample sizes, standard deviations and *P* values for primary outcomes at 6-month follow-up in the original and repeat trials

Variable	Original trial								Repeat trial							
	Control			Intervention					Control			Intervention				
	% or mean	SD	*n*	% or mean	SD	*n*	*p*	*d*	% or mean	SD	*n*	% or mean	SD	*n*	*p*	*d*
Fruit and vegetable intake																
Portions per day	5.72	4.98	512	5.61	4.89	495	0.708	−0.02	3.89	1.97	793	4.11	1.84	690	0.024	0.12
Physical activity																
Metabolic equivalent of task	3316.10	5143.79	526	3350.52	5144.16	513	0.914	0.01	3613.27	2578.07	788	3627.94	2578.97	671	0.932	0.01
Alcohol consumption																
Units in last 7 days	13.41	19.65	547	13.10	19.75	540	0.737	0.02	11.03	10.91	782	10.42	10.86	668	0.277	0.06
Smoking																
Current smoker	13.02	–	553	8.70	–	540	0.010	0.25	14.05	–	783	11.18	–	671	0.293	0.10

### Moderation analysis

Sex, nationality (UK vs. non-UK), and ethnicity (white vs. non-white) did not moderate the effect of the intervention on any of the primary outcome variables.

### Engagement

Of the 1,346 participants allocated to the intervention condition, 1,149 (85 %) completed the self-affirmation task. Considering engagement with the health messages, 973 participants (72 %) viewed a message for at least one behaviour, 672 (50 %) for at least two behaviours, 640 (48 %) for at least three behaviours, and 630 (47 %) for all four behaviours. Considering engagement with the planning tasks, 554 participants (41 %) formed an implementation intention for at least one behaviour, 479 (36 %) for at least two behaviours, 439 (33 %) for at least three behaviours, and 395 (29 %) for all four behaviours.

### Per-protocol analysis

To assess the effect of engagement with the intervention on the primary outcomes, per-protocol analyses were conducted that included all participants in the control condition (*N* = 799) and, for each health behaviour, only those participants in the intervention condition who completed the self-affirmation profile, viewed a health message and formed an implementation intention (*N* = 281 for fruit and vegetable intake, 297 for physical activity, 253 for alcohol consumption, and 238 for smoking). These analyses revealed that participants in the intervention condition who had engaged with the intervention reported consuming significantly more portions of fruit and vegetables, *F* (1, 1068) = 7.19, *P* = 0.007, than those in the control condition (mean = 4.23, 3.89; SD = 0.11 and 0.07, respectively). Like the primary analyses, the per-protocol analyses revealed no significant effect of the intervention on levels of physical activity, *F* (1, 1079) = 0.80, *P* = 0.371, units of alcohol consumed, *F*(1, 1030) = 1.30, *P* = 0.254, or current smoking status, *B* = −0.33, SD = 0.33, *P* = 0.332.

### Secondary outcomes

The intervention was found to have a number of significant effects on secondary outcomes (see Table 4). In particular, the intervention had a significant effect on smoking at university at 6-month follow-up, such that 37.16 % of participants in the control condition reported that they had smoked since starting university compared with only 30.70 % of participants in the intervention condition. In addition, the intervention had a significant effect on the biochemical marker of alcohol use (fatty acid ethyl esters) at 6-month follow-up, with lower levels of alcohol use observed among participants in the intervention versus control condition. The intervention also had significant effects on three social cognitive variables. Participants in the intervention condition had a more negative cognitive attitude towards binge drinking than participants in the control condition at 1-month follow-up. However, contrary to expectations, participants in the intervention condition had lower self-efficacy scores for fruit and vegetable intake and physical activity than participants in the control condition at 6-month follow-up. The effect of the intervention on all other secondary outcomes (e.g., recreational drug use, health status, BMI, health service usage) was non-significant.

**Table 4:** Estimated marginal means, percentages, sample sizes, standard deviations and p values for secondary outcomes

	1-month follow-up								6-month follow-up							
Variable	Control			Intervention					Control			Intervention				
	% or mean	SD	*n*	% or mean	SD	*n*	*p*	*d* (odds ratio)	% or mean	SD	*n*	% or mean	SD	*n*	*P*	*d* (odds ratio)
Fruit and vegetable intake																
Portions per day	3.63	1.84	691	3.84	1.95	595	0.041	0.11								
Physical activity																
Metabolic equivalent of task	3501.75	2348.35	690	3515.20	2348.97	579	0.919	0.01								
Alcohol consumption																
Units in the last 7 days	12.06	12.01	682	11.45	11.78	578	0.364	0.05								
Number of days binge drinking in previous 7 days (drinkers only)	1.02	0.90	508	0.98	1.03	421	0.547	0.004	0.99	0.95	564	0.97	0.87	473	0.674	0.02
Alcohol Use Disorders Identification Test									9.32	4.94	504	9.40	4.90	416	0.809	−0.02
Biochemical marker of alcohol consumption (fatty acid ethyl esters)									7.29	7.85	70	5.00	4.33	63	0.038	0.35
Smoking																
Has smoked	51.90	–	684	52.50	–	581	0.876	(1.01)	54.72	–	795	53.57	–	685	0.933	(0.96)
Smoked since attending university	27.34	–	684	26.16	–	581	0.723	−0.02 (0.97)	37.16	–	783	30.70	–	671	0.016	0.11 (0.81)
Current smoker	11.84	–	684	11.36	–	581	0.390	−0.03 (0.96)								
Cigarettes smoked per week (smokers only)	16.21	20.11	81	17.02	25.87	66	0.832	−0.04	19.87	28.19	74	18.30	21.75	46	0.749	0.06
Smoking marker (cotinine)									1.02	6.40	70	0.38	1.49	63	0.460	0.13
Smoking marker (nicotine)									2.52	11.94	70	2.91	17.96	63	0.867	0.03
Other outcomes																
EQ-5D-3 L																
Health index scores from EQ-5D-3 L (visual analogue scale)	0.92	0.26	663	0.91	0.24	559	0.907	−0.04	0.91	0.28	810	0.90	0.26	702	0.262	−0.04
Health index scores from EQ-5D-3 L (time trade off)	0.93	0.26	663	0.93	0.24	559	0.873	0.00	0.93	0.28	810	0.92	0.26	702	0.208	−0.04
EQ-5D-3 L visual analogue scale	78.61	13.97	669	79.47	14.07	569	0.285	0.06	79.16	12.98	763	78.98	13.07	657	0.791	−0.01
Recreational drug use																
Have taken recreational drugs (single sample count method)	4.84	8.83	708	4.00	9.61	608	0.450	0.11	23.18	11.18	586	21.78	12.00	493	0.556	0.04
Have taken recreational drugs (biochemical marker)									55.71	-	70	52.38	–	63	0.717	(0.88)
Body mass index																
Self-report	21.63	1.70	591	21.52	1.77	491	0.302	0.06	22.00	2.38	698	21.84	2.44	596	0.231	0.07
Objective									21.79	2.54	70	22.43	3.49	63	0.255	−0.20
Health service usage																
Times visited general practitioner in previous 6 months									1.48	2.31	532	1.48	0.11	449	0.973	0.00
General practitioner offered alcohol intervention									1.90	–	9	0.80	-	3	0.156	0.36
No alcohol intervention offered									98.1	–	463	99.2	-	394		(1.92)
Attended alcohol intervention									66.7	–	2	0.00	-	0	0.889	–
Did not attend alcohol intervention									33.3	–	4	100	-	2		–
Times visited accident and emergency department									0.13	0.46	532	0.11	0.42	446	0.439	0.05
Times admitted to accident and emergency department									0.15	0.38	57	0.08	0.32	42	0.319	0.20
Times required an ambulance									0.04	0.45	503	0.05	0.42	435	0.683	−0.02
Times admitted to hospital									0.05	0.23	525	0.05	0.21	444	0.828	0.00
Elective admissions to hospital									0.95	0.69	21	0.84	0.68	18	0.637	0.16
Non-elective admissions to hospital									0.36	1.88	210	0.37	0.62	17	0.964	−0.01
Other times visited hospital (not including above)									0.34	0.91	523	0.32	1.05	439	0.877	0.02
Social cognition variables																
Fruit and vegetables																
Affective attitude	5.96	1.28	658	5.94	1.18	560	0.823	−0.02	6.03	1.17	545	6.07	1.28	454	0.607	0.03
Cognitive attitude	6.78	0.77	656	6.75	0.71	559	0.380	−0.04	6.82	0.70	543	6.77	0.64	454	0.212	−0.07
Subjective norm	6.14	1.28	658	6.13	1.18	559	0.885	−0.01	6.26	1.17	545	6.21	1.07	455	0.495	−0.04
Descriptive norm	2.67	1.28	659	2.63	1.18	561	0.557	−0.03	2.72	1.17	545	2.69	1.28	455	0.644	−0.02
Self-efficacy	5.63	1.54	658	5.50	1.66	561	0.170	−0.08	5.83	1.40	545	5.57	1.49	456	0.005	−0.18
Perceived control	5.43	1.54	659	5.42	1.66	560	0.953	−0.01	5.63	1.63	544	5.56	1.49	456	0.479	−0.04
Intention	4.68	1.79	657	4.79	1.65	558	0.235	0.06	4.93	1.63	540	4.96	1.71	455	0.843	0.02
Planning	5.00	1.79	657	4.93	1.66	560	0.564	−0.04	5.14	1.63	544	5.14	1.71	455	0.998	0.00
Physical activity																
Affective attitude	5.65	1.54	659	5.61	1.66	560	0.680	−0.03	5.73	1.40	545	5.76	1.49	454	0.742	0.02
Cognitive attitude	6.80	0.77	658	6.77	0.71	561	0.539	−0.04	6.84	0.70	544	6.77	0.64	454	0.105	−0.10
Subjective norm	6.07	1.28	659	6.09	1.18	560	0.821	0.02	6.20	1.17	545	6.21	1.07	455	0.894	0.01
Descriptive norm	3.68	1.28	659	3.78	1.42	561	0.202	0.07	3.86	1.40	544	3.83	1.28	454	0.706	−0.02
Self-efficacy	5.68	1.54	658	5.57	1.42	561	0.198	−0.07	5.88	1.40	545	5.71	1.49	456	0.049	−0.12
Perceived control	5.60	1.54	658	5.59	1.66	561	0.886	−0.01	5.73	1.63	545	5.65	1.49	455	0.441	−0.05
Intention	5.34	1.80	660	5.43	1.66	560	0.364	0.05	5.32	1.87	545	5.47	1.71	455	0.158	0.08
Planning	4.98	1.80	658	5.00	1.89	560	0.902	0.01	5.22	1.87	544	5.19	1.71	455	0.788	−0.02
Binge drinking																
Affective attitude	3.17	1.80	659	3.08	1.89	560	0.379	0.05	3.02	1.87	545	3.03	1.71	455	0.944	−0.01
Cognitive attitude	1.82	1.03	658	1.68	0.95	561	0.019	0.14	1.68	0.93	543	1.68	1.07	454	0.998	0.00
Subjective norm	2.43	1.54	659	2.33	1.42	560	0.275	0.07	2.36	1.40	543	2.40	1.49	455	0.694	−0.03
Descriptive norm	5.63	1.28	659	5.60	1.42	561	0.616	0.02	5.57	1.17	545	5.71	1.28	455	0.071	−0.11
Self-efficacy	6.13	1.28	658	6.16	1.42	561	0.783	−0.02	6.20	1.40	545	6.15	1.28	456	0.574	0.04
Perceived control	5.68	1.80	659	5.76	1.66	561	0.400	−0.05	5.82	1.63	543	5.89	1.71	455	0.461	−0.04
Intention	3.38	1.80	658	3.24	1.89	558	0.210	0.08	3.28	1.86	543	3.19	1.92	455	0.434	0.05
Planning	4.87	2.05	657	4.79	2.13	560	0.478	−0.04	4.99	2.10	543	4.84	2.13	455	0.244	−0.07
Smoking																
Affective attitude	1.55	1.28	658	1.54	1.18	560	0.940	0.01	1.32	0.70	537	1.35	0.69	525	0.491	−0.04
Cognitive attitude	1.27	0.77	658	1.26	0.71	559	0.648	0.01	1.59	1.17	545	1.53	1.28	455	0.398	0.05
Subjective norm	1.31	0.77	658	1.30	0.71	559	0.854	0.01	1.35	0.93	545	1.35	0.85	455	0.940	0.00
Descriptive norm	4.01	1.54	658	3.98	1.42	561	0.709	0.02	4.04	1.40	545	4.16	1.49	455	0.169	−0.08
Self-efficacy	6.53	2.05	658	6.52	1.18	561	0.899	0.01	6.48	1.17	545	6.55	1.28	456	0.384	−0.06
Perceived control	5.46	2.05	659	5.33	1.89	561	0.272	0.07	5.52	1.86	543	5.54	1.92	454	0.864	−0.01
Intention	1.56	1.28	658	1.58	1.42	558	0.826	−0.01	1.67	1.40	545	1.59	1.28	455	0.322	0.06
Planning	5.86	2.05	658	5.79	1.89	560	0.524	−0.04	5.81	1.86	542	5.69	1.92	454	0.342	−0.06

## Discussion

This paper reports the results of a repeat randomized controlled trial of a theory-based online health behaviour intervention delivered during the transition from school to university. The original trial [16] was compromised by a number of study limitations that resulted in low levels of engagement with the intervention. As a result, a number of changes were instigated in the repeat trial to increase engagement, including a shorter baseline questionnaire (to reduce participant fatigue), the use of the LifeGuide open-source software platform [18] to deliver the intervention (to improve the experience of engaging with the intervention), and a streamlined modular structure (so that participants could access the intervention material more quickly). These changes were successful in increasing engagement with the intervention. Thus, 85 % of participants in the intervention condition completed the self-affirmation manipulation compared with 52 % in the original trial, 72 % accessed at least one health message (and 47 % accessed health messages for all four health behaviours) compared with the 35 % who accessed the health messages in the original trial, and 41 % formed at least one implementation intention (and 29 % formed implementation intentions for all four health behaviours) compared with 1 % in the original trial.

Despite increased engagement, the primary analyses indicated that the effect of the intervention on the targeted health behaviours at 6-month follow-up was non-significant, although the effect on fruit and vegetable intake approached significance. Moreover, a per-protocol analysis revealed that participants who engaged with the intervention reported consuming significantly more portions of fruit and vegetables at 6-month follow-up than participants in the control condition. The original intervention was found to have a significant effect on smoking status at 6-month follow-up, which was primarily due to preventing non-smokers from starting smoking at university. This effect was not replicated in the repeat trial, although the direction of the effect was consistent. In addition, secondary analyses indicated that significantly fewer participants in the intervention condition reported that they had smoked at university (including a puff or socially) than those in the control condition. The intervention was also found to have a significant impact on the biochemical marker of alcohol use at 6-month follow-up, with lower levels of alcohol observed among participants in the intervention than in the control condition. This effect was in the same direction as the non-significant effect of the intervention on alcohol consumption in the primary analyses. Notably, participants who provided a hair sample did not differ from the rest of the baseline sample in their health behaviour.

The effect sizes for the intervention on the primary outcomes in the repeat trial were comparable to those found in the original trial, although two differences approached significance. The effect size for intervention on fruit and vegetable intake was marginally larger in the repeat trial, whereas the effect size for smoking status was marginally larger in the original trial. Overall, the effect sizes found in both trials were very small [33] and, with the exception of smoking status in the original trial, smaller than those typically found in online health behaviour change interventions [8].

There are several potential reasons for the relatively weak effects obtained in the repeat trial (which also relate to the original trial). First, the intervention sought to target four health behaviours in a single intervention. Webb *et al.* [8] reported that online interventions that target several health behaviours typically have smaller effects (*d* = 0.12) than those that target a single health behaviour (*d* = 0.17). A focus on several health behaviours may dilute the effect of the intervention on individual behaviours if participants choose only to change a single health behaviour. For example, an intervention targeting multiple health behaviours might help some participants to increase their fruit and vegetable intake and others to increase their levels of physical activity. Second, although levels of engagement with the intervention were increased in the repeat trial, they were still relatively low. Again, this may have been due to the focus on several health behaviours. For example, while 72 % of participants in the intervention condition accessed at least one health message, only 47 % accessed health messages for all four health behaviours. Similarly, while 41 % formed at least one implementation intention, only 29 % formed implementation intentions for all four health behaviours. Furthermore, there was some evidence that engagement moderated intervention effectiveness, with the per-protocol analyses revealing a significant effect of the intervention on fruit and vegetable intake (although other effects remained non-significant). Third, the baseline sample recruited into the study reported engaging in the recommended health behaviours at a similar or greater extent than 16–24-year-olds in England [2, 3]. It is therefore possible that the lack of positive effects for the intervention could be due to a ‘ceiling effect’ at baseline. However, analysis of participants in the control condition revealed that consumption of fruit and vegetables decreased, and alcohol consumption and the number of current smokers increased, after starting university, which would negate this explanation for the null findings.^1^

A number of limitations should be noted. First, in line with the original trial and the study protocol [9, 16], the effect of the intervention on the primary outcomes was assessed using an intention-to-treat approach in which missing data at 6-months were imputed from the 1-month follow-up data by carrying the last observation forward. The use of this procedure has been criticized as it may introduce bias in the results (in either direction) and lead to overly narrow confidence intervals [32]. In particular, it assumes that students’ health behaviour would have remained stable from 1- to 6-month follow-up. Analysis of the control condition revealed changes in three of the four health behaviours between these two time points. However, as recommended by Altman [32], repeating the primary analyses without data imputation produced consistent results.

Second, the primary outcomes were assessed by self-report. To try to address this issue, we also sought to identify biochemical markers of alcohol and smoking behaviour by analyzing samples of hair provided by participants. Only 213 (8 %) of participants recruited into the trial participated in this aspect of the study. While highly selective, these participants were not found to differ from the rest of the sample on baseline measures of health behaviour. It should also be noted that there were no biochemical markers of fruit and vegetable intake, which precludes verification of the significant effect of the intervention effect found in the per-protocol analyses. Moreover, the fact that per-protocol analyses only included participants in the intervention condition who completed all intervention tasks (i.e., those who completed the self-affirmation profile, viewed a health message and formed an implementation intention) is likely to have introduced bias. As a result, the significant effect of the intervention on fruit and vegetable intake found in the per-protocol analyses should be treated with caution.

Third, although the response to the initial invitation emails was higher (48.1 % vs. 31.3 %) in the repeat trial (primarily due to the invitation emails being sent out a week earlier and the use of a shorter baseline questionnaire), recruitment into the study was still relatively low. This was despite the use of a number of techniques that have been shown to increase response rates to online surveys, including the use of incentives and reminders [34]. In addition, participation in the repeat trial was higher among women, non-UK students and younger students, which may limit the generalizability of the findings.

Fourth, attrition was higher in the repeat trial, with only 55.8 % of participants providing follow-up data (vs. 76.6 % in the original trial), and was higher in the intervention arm than the control arm, which may have been a consequence of the longer time that participants spent engaging with the intervention in the repeat trial. Participants who dropped out were found to have a lower BMI and stronger intentions to eat fruit and vegetables at baseline than participants who provided follow-up data. Participants who dropped out were also less likely to be British, white or female.

Fifth, the intervention used three theory-based techniques (i.e., self-affirmation, theory-based messages and implementation intentions), which were combined in a single intervention. Such a design precludes the identification of the active, or redundant, ingredients of an intervention. Future work could employ full factorial designs to assess the impact of different combinations of these techniques on health behaviour. There is some evidence that combining self-affirmation manipulations and implementation intentions has a beneficial effect on fruit and vegetable intake [35], although other research suggests that this combination may have a detrimental effect on physical activity [36].

## Conclusions

A repeat trial of an online theory-based health behaviour intervention for new university students found no significant effects on the primary outcome measures at 6-month follow-up, although three positive effects were observed in ancillary analyses. First, a per-protocol analysis showed a significant effect of the intervention on fruit and vegetable intake. Second, secondary analyses revealed that significantly fewer participants in the intervention condition had smoked while at university than in the control condition. Third, analysis of hair samples demonstrated significantly lower use of alcohol among participants in the intervention condition than in control condition. Nonetheless, the overall effect of the intervention on the targeted health behaviours was relatively weak. This may have been due, in part, to the focus of the intervention on multiple, rather than, single health behaviours. Future interventions targeting the health behaviour of new university students might focus on smoking behaviour, given the positive intervention effects in the original and repeat trials and the important impact that smoking has on health outcomes.

## Endnote

^1^ Analysis of participants in the control condition revealed significant effects of time on portions of fruit and vegetables consumed, *F* (2,932) = 30.17, *P* < 0.001, units of alcohol consumed, *F* (2,910) = 48.83, *P* < 0.001, and number of current smokers, Cochran’s *Q* (2) = 27.94, *P* < 0.001. Post-hoc analyses indicated that the consumption of fruit and vegetables decreased from baseline (mean = 4.41, SD = 1.92) to 1-month follow-up (mean = 3.70, SD = 1.90) and then increased from 1- to 6-month follow-up (mean = 3.98, SD = 2.07), alcohol consumption increased from baseline (mean = 6.72, SD = 9.79) to 1-month follow-up (mean = 11.99, SD = 14.72) and then decreased from 1- to 6-month follow-up (mean = 10.57, SD = 12.97), and the number of current smokers increased from baseline (3.08 %) to 1-month follow-up (5.45 %) and also from 1- to 6-month follow-up (9.00 %). Differences between all time points were significant for all three of the health behaviours. Physical activity levels remained stable over time, *F* (2,920) = 0.99, *P* = 0.37.

## Abbreviations

ANCOVA: analysis of covariance
AUDIT: Alcohol Use Disorders Identification Test
BMI: body mass index
